# E-Cigarette Quality Control: Impurity and Nicotine Level Analysis in Electronic Cigarette Refill Liquids

**DOI:** 10.1155/2020/3050189

**Published:** 2020-04-13

**Authors:** Ismail Bennani, Madiha Alami Chentoufi, Miloud El Karbane, Amine Cheikh, Mustapha Bouatia

**Affiliations:** ^1^Laboratory of Analytical Chemistry, Faculty of Medicine and Pharmacy of Rabat, University Mohamed V, 10000 Rabat, Morocco; ^2^Departement of Pharmacy, Faculty of Pharmacy, University Abulcasis, 10000 Rabat, Morocco

## Abstract

This work targets mainly the quality control of electronic cigarette liquids. It relies on an analytical control of a “32-product” sample made of several types of e-cigarette liquids taken from various supermarkets and tobacconist's offices in Morocco. All along this study, we made sure to check both the conformity of the nicotine level indicated in the packaging of each product and the existence of any other components inside the product, especially toxic or unknown impurities. The method used for this study is known under the name of high-performance liquid chromatography. For statistical analysis, we used Student's *t*-test for a single sample in order to analyze the relative differences between nicotine quantity reported in the product and the one measured during our experiment. Finally, we used linear regression test to determine the relationship between the nicotine level accuracy on the packaging and the level of toxic impurities in the products. The differences between the nicotine concentrations reported in the packages and the measured ones varied from −100% to +3.3%. The study showed that 31% of analyzed products have an accurate indication of the level of nicotine on the packaging. However, 47% of the studied products showed more than 20% difference between measure and packaging indication. In all analyzed samples, the level of impurities altered from 0 to 32.6%. Furthermore, the level of the nicotine breakdown products did not exceed 2% of the nicotine content in pretty much all of the samples. The actual nicotine content of electronic cigarette refill liquids is not always as precise as what is stated on the packaging; in addition to the level of impurities detected in several brands and that exceeds the European Pharmacopoeia standards, some may even present a risk of causing toxicological damage.

## 1. Introduction

Smoking has always been a major health problem. According to the WHO (World Health Organization), there are six million deaths each year all over the world that are due to tobacco [1]. Several diseases have been related to the use of cigarettes; most of them are either cardiac vascular (stroke, arterial disease), respiratory (chronic obstructive bronchitis), or neoplastic (lung cancer) [2, 3]. Tobacco, the main substance in cigarettes, is also known to be highly addictive which makes it the leading cause of death in the world [4]. It is estimated that one in two smokers will die from smoking [1].

Recently, a new concept has emerged called tobacco harm reduction [5]and which aim is to reduce tobacco consumption. It is based on the substitution of cigarette by a less harmful competitor product. In fact, it was considered by many experts as the way to move forward [6]. Several studies have focused on the contribution of alternative or substitute nicotine products in a harm reduction strategy [7–9]. During a study lead on a group of intensive smokers (more than 20 cigarettes per day), the use of alternative products allowed an increase in smoking cessation attempts [9]. However, from a consumer's perspective, current substitutes created from the issue of not delivering nicotine in the same way as cigarettes. This urged companies to develop a new product that can release nicotine in a similar way from cigarettes in terms of gesture and sensation. This is definitely the case of a popular recent product: the electronic cigarette or the e-cigarette [10].

Appeared in China in the early 2000s, this device has, since 2010, made a significant progress. Its main characteristic is to allow the delivery of nicotine via a vapor composed of a mixture of propylene glycol or glycerin in the alcohol. As they do not initially allow a very good absorption of nicotine [10], e-cigarettes have evolved technologically to rechargeable models delivering up to more than half of the nicotine present in the cartridges which were highly appreciated by users [11, 12]. In several studies, the majority of e-cigarettes users said that it helped them reduce or even stop smoking altogether [13]. However, from the scientific community perspective, its use remains cautious [14].

The fluids of the electronic cigarette often contain a mixture of nicotine and other constituents such as minor alkaloids, acetaldehyde, and propylene glycol [15–17].

Numerous studies have shown that a high dose of nicotine increases the intracranial self-stimulation (ICSS) and triggers a reduction in the acute anhedonic adverse effects of nicotine when administered in electronic cigarette liquid. Since these effects of nicotine may limit its absorption, it can be expected that its reduction will increase e-cigarette consumption [18]. However, certain studies found no difference in intravenous self-administration of nicotine alone in comparison with in liquid cigarettes [17].

Whatever the interpretation of these results, they show at least that the e-cigarette liquid contains active ingredients of constituents other than nicotine (according to ICSS measure). In order to understand the relative contribution of the central nervous system effects of nicotine components and nonnicotine components in the risk of the e-cigarette misuse, a study on a rat population showed the acute effects of electronic cigarette liquid on ICSS [19].

From a regulatory point of view, the current legislation in the world for e-cigarettes varies considerably across countries. The two main reasons are often the presence or absence of nicotine in the product and the presentation of e-cigarettes as a useful therapeutic product to reduce smoking. And since this last point is crucial, therapeutic products must comply with regulations that require rigorous quality control with advanced toxicity analysis [20].

In light of increasing efforts to improve the quality of tobacco harm reduction products, we analyzed a sample of e-cigarettes using an HPLC method in order to compare the actual nicotine levels with the ones stated on the packaging and to observe the possible presence of other components in the product.

## 2. Methods and Materials

### 2.1. Methods

After determining nicotine levels and comparing their conformity with the values mentioned on the packaging labels, the nicotine breakdown products and the unidentified impurities have been quantified referring to the applicable European Pharmacopoeia (10.0).

### 2.2. Materials and Reagents

First, we used a nicotine reference standard (99.6% purity) from Chans Flavor Company (China). It is compliant with both the European and US Pharmacopoeia. Then, we analyzed 32 samples that were selected from 25 different brands. Some of these brands presented different strength and flavor which allowed us to reach 32 samples.

All reagents that were used for the preparation of the mobile phase are chemical grade reagents except acetonitrile which is of HPLC grade. We purchased acetic acid, ammonia, and acetonitrile from Merck Chemicals, Germany, and we used purified water as the diluting solvent.

As for the liquid samples of the e-cigarettes, we got these from different local tobacco shops or supermarkets, and all of them were stamped as European or US products.

### 2.3. Instrument

We used the chromatographic systems constituted by a Waters 2695 pump, auto sampler, and Waters 2998 photodiode array detector, while Empower Software and Spectra Manager software data registration was used for all absorbance measurements. We also used TotalChrom software for data acquisition (USA) and Mettler Toledo scale for all weighing.

### 2.4. Chromatographic Conditions

The chromatographic conditions are described in the current European Pharmacopoeia:

1. Column: size: *l* = 0.15 m, Ø = 4.6 mm; stationary phase: end-capped polar-embedded octadecylsilyl amorphous organosilica polymer *R* (5 *μ*m)
2. Mobile phase: mobile phase A: 900 mL of water added to 25 mL of 60 g/L solution of acetic acid *R* and then added to 6 mL of concentrated ammonia. pH adjustment to 10.0 with dilute ammonia or dilute acetic acid and dilution to 1,000 mL with water; mobile phase B:acetonitrile
3. The mobile phase was filtered through a 0.45 *μ*m Millipore filter and degassed under vacuum before use
4. Dilution medium: distilled water
5. Flow rate: 1.0 mL/min
6. Detection: spectrophotometer at 254 nm
7. Injection volume: 20 *μ*L
8. Temperature: ambient temperature
9. Identification of impurities: in order to identify the peaks due to impurities *A*, *B*, *C*, *D*, *E*, *F*, and *G*, the chromatogram supplied with nicotine for system suitability and the chromatogram obtained from reference solution both should be used
10. Relative retention with reference to nicotine (retention time = about 17.8 min): impurity *E* = about 0.3; impurity *C* = about 0.55; impurity *F* = about 0.7; impurity *A* = about 0.8; impurity *D* = about 0.86; impurity *G* = about 0.9; and impurity *B* = about 1.6.
11. Impurity *A*: (2S)-1,2,3,6-tetrahydro-2,3′-bipyridyl (anatabine); impurity *B*: 3-(1-methyl-1H-pyrrol-2-yl) pyridine (*β*-nicotyrine); impurity *C*: (5S)-1-methyl-5-(pyridin-3-yl) pyrrolidin-2-one (cotinine); impurity *D*: 3-(4,5-dihydro-3H-pyrrol-2-yl) pyridine (myosmine); impurity *E*: (1RS, 2S)-1-methyl-2-(pyridin-3-yl) pyrrolidine 1-oxide (nicotine N′-oxide); impurity *F*: 3-[(2S)-pyrrolidin-2-yl]pyridine (nornicotine); and impurity G: 3-[(2S)-piperidin-2-yl] pyridine (anabasine)

### 2.5. Statistical Analysis

We calculated the mean nicotine concentration of each sample, the standard deviation, and the mean difference between the actual and reported values using the Student's *t*-test.

We then established the relationship between nicotine concentrations and the rate of impurities using a linear regression test. The linear regression test was performed to determine the relation between the variation of the nicotine level and the impurity rate in the e-cigarette liquid. For all tests, we used SPSS (V. 24.0) software for the statistical analysis (see appendix 2 for more details).

## 3. Results and Discussion

We analyzed 32 samples from different brands of electronic cigarette liquids. The results are presented in Table 1. The comparison of the labeled and determined nicotine concentrations in all the e-liquids analyzed in the study is made by Student's *t*-test for the unique sample (Table 2).

All in all, the differences between measured and reported nicotine concentrations ranged from -100% to 3.3%. 10 tested samples (31%) showed a similarity between the labeled and the detected nicotine concentration, while 18 samples (47%) showed differences (higher than 20%) between these. Traces of nicotine were found in two samples (samples 15 and 22), and no nicotine trace was detected in the two others two, even if it is mentioned in the package.

**Figure 1:**
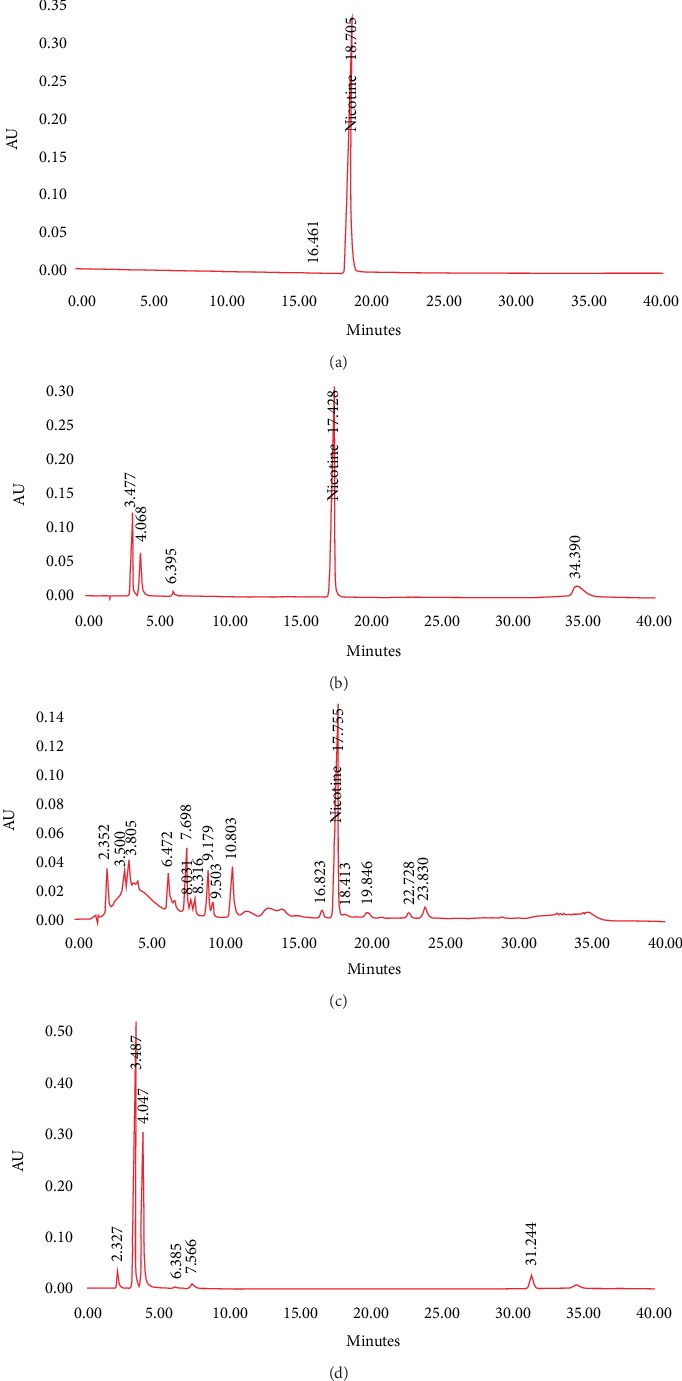
Example of chromatograms: (a) chromatogram A, (b) chromatogram B, (c) chromatogram C, and (d) chromatogram D.

Figure 1 shows examples of chromatograms: chromatogram (A): reference solution; chromatogram of sample 2 (B): the chromatogram of the sample had many nicotine-related peaks; chromatogram of sample 1 (C): the chromatogram showed many known breakdown products, along with many other peaks; and chromatogram of sample 3 with nicotine free (D). The unidentified peaks found (chromatogram C) are not related to nicotine and may be related to flavorings or other excipients (Figure 1).

Table 1 shows the concentration of nicotine-related impurities and unknown impurities expressed as a percentage of the surface for nicotine. Quantification of known breakdown products and unidentified impurities related to nicotine were compared with the surface of the nicotine spike in the same samples. In all analyzed samples, the area of impurities accounted for between 0 and 32.6%, but for most samples, the level of the nicotine breakdown products did not exceed 2% of the nicotine content. Nicotine-N-oxide, anatabine, myosmine, and anabasine were the most common substances found. Samples 8 and 11 containing only nicotine were the cleanest of all samples, with almost no nicotine-related substances or unknown impurities. Empty cells in the table show that the substances were not present in these samples (Table 1).

Table 3 shows the multiple linear regression of the relative differences in nicotine levels on nicotine-related impurities and total impurities.

The statistical analysis based on the linear regression test showed that there is no relation between the difference of nicotine level (between the value found and the value mentioned in the packaging) and the impurity rate in the liquid (*p* > 0.05).

We analyzed 32 samples of several brands of refill liquids marketed in Morocco for the electronic cigarette and found that the contents of nicotine breakdown products and nicotinic impurities represented between 0% and 11% of the nicotine content, but for the majority of e-liquids, the level was between 1 and 4%. Nicotine-N-oxide, myosmine, anatabine, and anabasine were the most commonly degrading or nicotine-related substances in the solutions. Cotinine and n-oxide-nicotine are also created during the metabolism of nicotine. These metabolites are both less potent and less toxic than nicotine itself [21, 22], and their presence in e-liquids' authorized levels could therefore be acceptable.

However, the presence of high levels of other degradation products or unjustified impurities may have serious toxicological implications which will present additional risk to the users. Several studies have shown nootropic and antipsychotic effects of nicotine degradation products on animal models [23–25].

Our analysis showed not only the differences in quality between the brands but also the differences within the same brands [22]. In the same context, L. Ponzoni et al. demonstrated that e-cigarette vapor induces neurochemical, physiological, and behavioral alterations related to dependence; they also confirmed that compounds other than nicotine contribute to this addiction [26].

The origin of nicotine and its manufacturing process remain difficult to determine based on these data. Half of the liquids analyzed contained up to five times the maximum amount of impurities mentioned in the European Pharmacopoeia [27]. Only ten among the thirty-two products were aligned to European Pharmacopoeia recommendations.

High levels of nicotine-related impurities suggest that this oxidative degradation of nicotine occurred either during the manufacture of the ingredient or during the manufacture of the final liquids or because of an unstable formulation. For a high-quality product, it is essential to use high-quality raw materials and that the composition of the product is stable and nonreactive.

The statistical analysis by the linear regression test showed that there is no relation between the difference of the nicotine level (between the value found and the value mentioned in the packaging) and the impurity rate in the liquid, which can eliminate oxidative degradation as a major source of impurities and may incriminate other sources.

The impurities not linked to nicotine can be explained by undesirable interactions with the material used for packaging and its quality or improper handling of manufacturing and storage but also their conditions. The quality and origins of the flavors can be linked to this case as well [22].

Aroma is a known parameter that affects the stability of products. For example, nicotine is easily oxidized by common substances found in mint, vanilla, and fruit flavors [28].

The conditions of storage and conservation are also incriminated and can also be responsible for many unknown impurities. A recent study has shown that nicotine impurities increase under long-term storage conditions especially n-nicotine, cotinine, myosmine, and nornicotine [29].

Compared to other studies [22, 30, 31], our results indicate that the discrepancy between the measured and reported nicotine concentrations is much higher than the one previously reported. In addition, there is a higher level of impurities and that varies by its country's origin. We suspected that the products destined to the developing country may be different from the products marketed in industrialized countries, given the regulatory absence and the lack of rigorous control of these products, which may have unknown origins, especially with the high activity of trading counterfeit and contraband products. Statistical analysis using the linear regression test consolidates this reasoning and confirms that there are sources of impurities other than nicotine degradation.

## 4. Conclusion

In our study, more than half of the e-cigarette liquids analyzed contained acceptable levels of impurities related to nicotine degradation, but the nicotine content was very different from one product to another. However, some brands had levels of impurities above accepted limits for pharmaceuticals standards.

The processes of manufacture and importation of these products must be controlled, in particular, the excipients used, and the quality control tests and procedures should be implemented too.

The regulatory situation is widening in most countries and is still questionable even in developing ones. The manufacturers and liquid dispensers of e-cigarettes are not as much controlled by regulatory authorities as for controlling drugs. For some brands of these liquids at least, the manufacturing or control process is likely to be below the standards required for nicotine-based drugs.

Since the market of electronic cigarettes has largely developed outside of an appropriate regulatory framework, some manufacturers and suppliers apparently lack basic know-how security, and most do not provide information about their products and manufacturing processes. However, no country currently regulates e-cigarettes and their liquids as drugs. On the contrary, they are regulated as tobacco products or consumer products. The success and development of e-cigarettes are challenging the current regulatory regime, which allows and regulates nicotine only in tobacco and nicotine medications. This situation requires a discussion about the place of nicotine in our society and a review of the nicotine legislation in all products.

## Figures and Tables

**Table 1:** Amount of impurities related to the degradation of nicotine and unidentified impurities expressed as a percentage of the nicotine content.

Sample	Impurities (%)∗								Total
	Cotinine	Nornicotine	Anatabine	Myosmine	Anabasine	Beta-nicotyrine	Nicotine N-oxide	Unknown impurities	
**S1**		0.48	0.72	0.17		0.1		24.5	**25.97**
**S2**	0.46	8.8		1.47			0.33	12.5	**23.56**
**S3**									**0**
**S4**				0.33			0.36	1.2	**1.89**
**S5**	0.33							0.2	**0.53**
**S6**	1.21							1.2	**2.41**
**S7**					0.4			1.98	**2.38**
**S8**								0.4	**0.4**
**S9**									**0**
**S10**								1.1	**1.1**
**S11**									**0**
**S12**	0.1	0.45		0.24		0.06	0.3	10.1	**11.25**
**S13**	0.09	0.3			0.4	0.1	0.22	2.7	**3.81**
**S14**	0.7		0.32	0.56	0.23		0.16	1.8	**3.77**
**S15**		2.83		1.01				1.3	**5.14**
**S16**	1.49			1.2	1.83	0.5		15.4	**20.42**
**S17**								6.2	**6.2**
**S18**	1.68	0.86	0.23					20.6	**23.37**
**S19**	1.23	0.72	0.27	0.2			0.06	30.1	**32.58**
**S20**	1		0.76		1.48			19.2	**22.44**
**S21**		0.16						7.4	**7.56**
**S22**	0.24		0.18	0.34	0.23		0.08	1.6	**2.67**
**S23**	0.35	0.12		0.32			0.24	2.1	**3.13**
**S24**	1.12		0.45	0.21			0.08	1.1	**2.96**
**S25**		0.14	0.32		0.3			0.4	**1.16**
**S26**	0.8	0.12	0.8			0.1	0.3	0.67	**2.79**
**S27**	0.5		0.06		0.3			1.3	**2.16**
**S28**	0.05				0.07				**0.12**
**S29**		0.15					0.12	0.23	**0.5**
**S30**	0.65	0.13				0.2		0.12	**1.1**
**S31**				0.33			0.16	1.2	**1.69**
**S32**	0.31				0.08			0.2	**0.59**

*∗* Impurity rate expressed in percentage of nicotine content.

**Table 2:** Comparison of labeled and determined nicotine concentrations in 32 commercial liquids of e-cigarette (*n* = 32).

Sample	Country origin	Liquid flavor	Labeled nicotine concentration (mg/mL)	Founded nicotine concentration (mg/mL) mean ± SD (*n* = 3)	Relative difference (%)	Significant difference*∗* (*p* < 0.05)
**S1**	USA	Tobacco	6	3.8	−36.67	Yes
**S2**	USA	Cookies	12	11.2	−6.67	Yes
**S3**	USA	Cherry	3	0	−100.00	Yes
**S4**	France	Blueberry	6	5.94	−1.00	No
**S5**	France	Strawberry	12	11.75	−2.08	No
**S6**	France	Mint	12	9.3	−22.50	Yes
**S7**	Spain	Fruit juice	3	2.91	−3.00	No
**S8**	France	Apple mint	6	5.4	−10.00	Yes
**S9**	USA	Honey	3	0	−100.00	Yes
**S10**	Germany	Strawberry	6	3.7	−38.33	Yes
**S11**	France	Coffee	12	4.1	−65.83	Yes
**S12**	USA	Green apple	6	5.9	−1.67	No
**S13**	France	Raspberry	6	2.2	−63.33	Yes
**S14**	USA	Strawberry	24	20.5	−14.58	Yes
**S15**	—	Mint	3	0.3	−90.00	Yes
**S16**	France	Tobacco	6	5.92	−1.33	No
**S17**	Spain	Honey	3	3.1	3.33	No
**S18**	—	Strawberry	6	1.6	−73.33	Yes
**S19**	France	Cherry	11	10.9	−0.91	No
**S20**	USA	Menthol	12	11.1	−7.50	Yes
**S21**	China	Banana	6	5.8	−3.33	No
**S22**	UK	Tobacco	3	0.2	−93.33	Yes
**S23**	France	Orange	12	9.1	−24.17	Yes
**S24**	China	Cookies	6	2.3	−61.67	Yes
**S25**	USA	Orange	11	10.5	−4.55	No
**S26**	USA	Blueberry	3	2.96	−1.33	No
**S27**	Germany	Strawberry	12	8.9	−25.83	Yes
**S28**	Belgium	Tobacco	6	5.5	−8.33	Yes
**S29**	China	Fruit juice	3	2.2	−26.67	Yes
**S30**	France	Green tea	6	5.6	−6.67	Yes
**S31**	China	Honey	3	2.1	−30.00	Yes
**S32**	USA	Tobacco	6	5.2	−13.33	Yes

*∗* Statistical significant difference (*p* < 0.05) between mentioned and detected nicotine level by *t*-test.

**Table 3:** The multiple linear regression of the relative difference in nicotine levels on nicotine-related impurities and total impurities.

	Standardized coefficients *β*	*p*
Nicotine-related impurities	−0.107	0.559
Impurity total	−0.171	0.349

## Data Availability

The data obtained after this quantitative and qualitative analysis which are used to support the conclusions of this study are all included in the article.
